# Was the Mare Pregnant?

**Published:** 1884-01

**Authors:** Joseph H. Miller


### II.—WAS THE MARE PREGNANT?

BY JOSEPH H. MILLER, V.S.

On the 28th of June a German called at my office, stating that he had a bay mare nine years old which had been suffering some time from *viser fluss*, or, in our language, leucorrhœa. I prescribed the usual remedies. In about ten days he returned, stating that the discharge was more abundant and very offensive. I gave as my opinion that the case was more serious than that of simple leucorrhœa, and went to see the mare at the stable. I found her very much reduced in flesh, the coat staring, legs swollen and a large tumor on the breast painful to the touch.

On making a vaginal examination I found the os uteri slightly dilated. I then introduced within the os a pill of hard extract of Bellodonna and waited an hour. I next dilated the os so as to allow the introduction of my three fingers, and labor pains commenced. At the expiration of an hour the uterus had expelled three gallons of a creamy-looking and offensive matter. I then cleaned out the cavity of the uterus with warm water and ordered daily injections of warm water, holding in solution carbolic acid in the proportion of 1 to 100, and small doses of ergot internally. On the third day I substituted sulphate of zinc injections for the others, using a solution of the strength of one drachm to the pint. I also ordered:

℞

Aloes, ʒvi.

Hg. submur. ʒi.

Zinziber. ʒii.

M. Given in a ball.

Afterwards the following:

℞

Fer. sulph.

Pot. nitrat.

Pot. bi-tart.

Gentian, āā, ʒi.

Sol. Fowler, ℥i.

M. In a ball morning and evening.

In six weeks the mare was so far improved as to be able to do her accustomed work—hauling milk to the factory.

Now as to the diagnosis. My opinion is, that as the mare was put to a horse a year previous and became pregnant, the fœtus died at the third or fourth month and underwent decomposition. Will the members of the veterinary profession be kind enough to give their opinion of the case in the pages of the JOURNAL?

[When the fœtus dies the symptoms of pregnancy are arrested. Milk sometimes appears in the mammaries; hæmorrhages from the uterus may occur, or they may not.

If the liquor amnii is not discharged it is absorbed, and the contents of the uterus either macerate or become mummified. If the membranes remain entire, the process undergone by the uterine contents is that of mummification.

It is only when germs are admitted and generally after the rupture of the bag of membranes that putrefaction and maceration take place, and the more or less complete dissolution of the ovum.

The uterus steadily diminishing in size, and almost invariably before the full term of pregnancy would have been reached, the ovum is expelled. When it is expelled you have a mass in a state of mummification nearly dry, of a dirty brown color, and the fœtus and membranes are concealed, being rolled up in the placenta.

Would consider it from the history given to be a case of uterine hæmatocele.—EDS.]

